# Texture tomography, a versatile framework to study crystalline texture in 3D

**DOI:** 10.1107/S2052252524006547

**Published:** 2024-07-24

**Authors:** M. P. K. Frewein, J. Mason, B. Maier, H. Cölfen, A. Medjahed, M. Burghammer, M. Allain, T. A. Grünewald

**Affiliations:** a Aix Marseille Univ, CNRS, Centrale Med Institut Fresnel,Marseille France; b University of California, Davis, California, USA; c University of Konstanz, Konstanz, Germany; d European Synchrotron Radiation Facility, Grenoble, France; ESRF, France

**Keywords:** *TexTOM*, computational modeling, nanostructure, computed tomography, WAXS

## Abstract

Crystallographic texture is a key organization feature of many technical and biological materials. In these materials, especially hierarchically structured ones, the preferential alignment of the nano constituents heavily influences the macroscopic behavior of the material. To study local crystallographic texture with both high spatial and angular resolution, we developed *Texture Tomography* (*TexTOM*). This approach allows the user to model the diffraction data of polycrystalline materials using the full reciprocal space of the crystal ensemble and describe the texture in each voxel via an orientation distribution function, hence it provides 3D reconstructions of the local texture by measuring the probabilities of all crystal orientations. The *TexTOM* approach addresses limitations associated with existing models: it correlates the intensities from several Bragg reflections, thus reducing ambiguities resulting from symmetry. Further, it yields quantitative probability distributions of local real space crystal orientations without further assumptions about the sample structure. Finally, its efficient mathematical formulation enables reconstructions faster than the time scale of the experiment. This manuscript presents the mathematical model, the inversion strategy and its current experimental implementation. We show characterizations of simulated data as well as experimental data obtained from a synthetic, inorganic model sample: the silica–witherite biomorph. *TexTOM* provides a versatile framework to reconstruct 3D quantitative texture information for polycrystalline samples; it opens the door for unprecedented insights into the nanostructural makeup of natural and technical materials.

## Abstract 2

The crystallographic texture is a key feature of crystalline organization in materials, yet no technique exists to locally characterize complex textured materials in 3D. In this manuscript, we present *Texture Tomography* (*TexTOM*) as a computational tool to provide full 3D texture information from X-ray diffraction measurements.

## 1. Introduction

The properties of many materials rely on their arrangement on the nano- and crystal structural level. Though this organization has shown to be of great importance for a wide host of both technical and biological materials such as concrete, steel or bone, wood, shells, and tendons to name but a few examples, its actual characterization poses a problem in the current day. The challenge for successful characterization during *in situ* and *operando* studies is to enable a high spatial and angular resolution while maintaining a large field of view and ideally providing a non-destructive imaging modality. Though electron microscopy based techniques boast impressive spatial resolution and with focused-ion beam tomography supply the possibility of 3D characterization, investigations are restricted to destructive sampling and in most cases *in vacuo* operation. X-rays however lend themselves to the task as they easily penetrate millimetres, even centimetres, in the case of hard X-rays for most technical materials. Recent advances in nanofocusing (Niese *et al.*, 2014 ▸) have enabled routine operation with beam sizes of 50 nm or less. The advent of fourth-generation synchrotron sources such as MaxIV (Tavares *et al.*, 2018 ▸) or ESRF-EBS (Raimondi *et al.*, 2023 ▸) has further unlocked the potential for *in situ* studies due to the impressive boost in flux that these machines deliver.

X-ray-based tomography methods have a long history of successful materials characterization. Following the routine implementation of full-field X-ray tomography and phase tomography (Paganin & Pelliccia, 2021 ▸), X-ray holo tomography (Cloetens *et al.*, 1999 ▸) and ptychographic tomography (Dierolf *et al.*, 2010 ▸) have enabled spatial resolution on a single-digit nanometre scale. The coupling of tomography and X-ray diffraction further opened the possibility to obtain orientation information, which is of high interest for polycrystalline materials with a non-random orientation distribution of crystallites. This property is called the ‘crystallographic texture’ (Kocks *et al.*, 2000 ▸). A whole class of 3D techniques exists to obtain local orientations of crystals from the position of Bragg peaks such as X-ray Bragg ptychography (Godard *et al.*, 2011 ▸), Bragg CDI (Williams *et al.*, 2003 ▸), 3D-XRD (Poulsen *et al.*, 2001 ▸) or DFXM (Simons *et al.*, 2015 ▸). All these techniques boast impressive angular resolution given, however, that the diffraction patterns show clearly distinguishable Bragg reflections. Hierarchically structured materials such as biominerals (*e.g.* bone, tendon, shell, cuticle), but also technical ceramics and some deformed metals, can be composed of a high number of crystallites whose crystal axes are locally distributed around a common mean orientation. This leads to azimuthally overlapping diffraction peaks and yields images resembling powder diffraction patterns with azimuthal variations in intensity, which are no longer possible to describe by a model that specifically addresses each individual crystallite or grain.

Another way to approach the problem is to consider first scattering tomography without orientation information, *e.g.* diffraction tomography approaches (Stock *et al.*, 2008 ▸; Bleuet *et al.*, 2008 ▸) or SAXS tomography (Schroer *et al.*, 2006 ▸). All these approaches assume a random orientation of the sample crystalline or nanostructural feature. Reconstructing directions based on nanostructural orientation required new ways to probe the reciprocal space and a parametrization for the orientation information, first shown by Georgiadis *et al.* (2015 ▸) for serial 2D slices. This lead to the development of tensor tomography, for which the seminal papers of Liebi and coworkers (Liebi *et al.*, 2015 ▸, 2018 ▸; Schaff *et al.*, 2015 ▸) have shown approaches to reconstruct orientation tensors from the small-angle scattering signal of nanostructures (SASTT), which have since found a wide host of scientific applications. The technique has also been extended into the wide-angle regime by Grünewald *et al.* (2020 ▸) for investigating Bragg peaks and used to study material properties of cartilage (Mürer *et al.*, 2021 ▸), tendon (Silva Barreto *et al.*, 2024 ▸), nervous tissue (Georgiadis *et al.*, 2021 ▸) and bone (Grünewald *et al.*, 2023 ▸), to give a non-exhaustive overview. Recent developments in the field have seen the introduction of more flexible reconstruction approaches that aim to overcome some of the isotropy and sampling assumptions of the original approach as well as improving the reconstruction speed (Gao *et al.*, 2019 ▸; Nielsen *et al.*, 2023 ▸). In particular, Nielsen *et al.* (2023 ▸) have presented significant performance improvements as well as an enhanced model flexibility by introducing band-limited spherical functions for the reconstruction.

All the aforementioned methods aim to reconstruct features in the diffraction pattern by modeling their intensity directly in reciprocal space. By reconstructing the position of a Bragg peak in 3D reciprocal space, for example, they obtain the preferred direction of a lattice plane or axis of a crystal. This leaves, however, one rotational degree of freedom to the orientation of the crystal, which could be obtained by independent reconstructions on several peaks and subsequent relation to the full crystal orientation tensor, analogous to the work of Johannes *et al.* (2020 ▸). However, the approach neglects the interconnection between the positions of the Bragg peaks during the reconstruction, given by the crystal symmetry, which in many cases is *a priori* well known. Motivated by classical texture analysis, a full implementation of the orientation distribution function (ODF) (Bunge, 1982 ▸) is used to tackle this problem. This means that we directly model the real space texture by assigning a probability to each unambiguous orientation of the crystal. The ODF fully determines the position of all Bragg peaks at once, allowing the reconstruction of full diffraction patterns with a single model.

Orientations of 3D objects have three degrees of freedom, which are traditionally described by Euler angles. A framework using a series expansion of generalized spherical harmonics (Bunge & Roberts, 1969 ▸; Roe, 1965 ▸) is often used to build the ODF and finds it use in state-of-the-art software packages (Hielscher & Schaeben, 2008 ▸). The use of spherical harmonics ensures a low number of adjustable parameters while providing the flexibility to model probabilities all possible orientations. Other approaches building on alternative ODF implementations and direct inversion strategies (Bachmann *et al.*, 2010 ▸; Lutterotti *et al.*, 1997 ▸; Matthies & Vinel, 1982 ▸) exist, but are geared more towards sharp textures usually obtained in metals and geological samples and are not reviewed here in detail. There are however shortcomings to the Euler angle parametrization, such as the degeneracy of orientations (Bunge, 2013 ▸; Robinson, 1958 ▸; Wigner, 2012 ▸), a distortion of the metric tensor (Morawiec, 1990 ▸) and singularities in the equations of motion (Robinson, 1958 ▸). To overcome these problems, we use a 3D harmonic expansion, which employs an axis–angle rotation parametrization to describe orientations (Mason & Schuh, 2008 ▸). This framework of hyperspherical harmonics (HSH) offers additional advantages such as computationally efficient rotation operations and the possibility of symmetrization according to the proper point group, which drastically reduces the number of parameters and opens the possibility to use only the fundamental zone of the orientation space.

Coupling this versatile model for describing crystallographic textures to tensor tomography, we are presenting *Texture Tomography* (*TexTOM*) as a computationally efficient framework to reconstruct full crystallographic texture information in 3D, based on scanning X-ray diffraction patterns. This lays the foundation for quantifying local texture by ODFs using the full information available in the diffraction patterns simultaneously. The outlined method will be described in detail in terms of its mathematical underpinning and the actual implementation of the code in Python. Numerical simulations to benchmark the performance of the method will be shown alongside the first experimental characterization of a helicoidal silica–witherite biomorph as an example of a hierarchically textured material.

## 2. Materials and methods

### 2.1. Biomorph sample

Silica–witherite biomorphs are a hierarchically structured, polycrystalline material. They are generated by the precipitation of barium carbonate in silicate-rich media at elevated pH (Garcia-Ruiz, 1985 ▸; Noorduin *et al.*, 2013 ▸). During the formation crystalline witherite (BaCO_3_) nanorods are formed and embedded in a silica matrix. Together, the biomorphs can take a variety of complex, curved shapes. The exact process governing the final shape is not yet fully understood (Kellermeier *et al.*, 2012 ▸). By modifying the properties of either the crystalline fraction (Holtus *et al.*, 2018 ▸) or functionalizing the silica matrix (Helmbrecht *et al.*, 2020 ▸; Opel *et al.*, 2016 ▸, 2020 ▸) new functionalities can be added, making biomorphs an attractive material system from a materials chemistry point of view. The biomorph sample employed here was produced by a one-pot co-precipitation method (Opel *et al.*, 2015 ▸). For this, in each field of a six-field well plate 5 ml of a 10 m*M* barium chloride solution were added to 5 ml of a 16.8 m*M* sodium *meta* silicate solution. The starting pH was adjusted to 11 using 1 m*M* NaOH. The gradual diffusion of atmospheric CO_2_ into the solution then yielded silica–witherite biomorphs of varying shapes at the bottom of the well plate. After 15 h the residual solution was removed and the structures were subsequently washed with water and ethanol before they were carefully detached from the bottom of the wells using a silicone brush. After transferring the biomorphs to a centrifuge vial using a pipette they were sedimented in a centrifuge using 9000 rpm for 10 min. After decanting the supernatant the structures were dried. For the synchrotron experiments, an ∼60 µm-long piece with the desired helicoidal shape was mounted on a thin (∼10 µm) glass capillary with epoxy glue.

### 2.2. Synchrotron experiments

For the experimental characterization, X-ray diffraction experiments were carried out at the ESRF-EBS beamline ID13 EH3 nanobranch. A 15.2 keV X-ray beam was produced by a channel-cut Si(111) monochromator and pre-focused by a set of compound beryllium lenses onto the final focusing optics, a set of multi-layer Laue lenses (MLLs), producing a beam of 300 × 300 nm with a flux of 10^12^photons s^−1^ on the sample position. The sample was mounted on a custom-designed goniometer (Grünewald *et al.*, 2020 ▸) based on Smaract actuators and scanned by a piezo stage. The diffraction signal was recorded with an Eiger X 4M and at an 157.78 mm sample-to-detector distance. The primary beam was blocked by a 500 µm lead beamstop. The setup gave access to the usable *q*-range 0.5–32 nm^−1^ and detector edges extending up to 40 nm^−1^. For each projection, the full sample was scanned with a step size of 500 nm in a continuous scanning mode and an adaptive field of view that enabled us to catch the full sample (maximum size 90 × 70 µm) while avoiding excessive air regions around the sample. Diffraction patterns were recorded with 2 ms exposure time. Subsequently, the sample was rotated around the *z*′ axis and tilted around the *y*′ axis. A total of 260 projections were collected for 10 tilt angles between 0 and 45°. At the 0° tilt angle, rotation angles were collected between 0 and 180° for all other tilt angles between 0 and 360°. The number of rotation angles in every tilt angle was reduced by a factor of 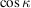. In total, the data acquisition took 6 h (with motor movement overheads). The total time of data acquisition was 3.6 h (6.5 Mio. diffraction patterns × 0.002 s). The dose deposited on the sample was calculated according to Howells *et al.* (2009 ▸) as follows: 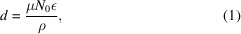
where μ = 269.58 cm^−1^ is the linear absorption coefficient for BaCO_3_ at 15.2 keV, *N*_0_ = 2.3040 × 10^−15^photons m^−2^ is the incident flux per unit area, ε = 2.4370 × 10^−15^ J is the photon energy and ρ = 4.3 g cm^−3^ is the mass density. Thus, the dose imparted on the sample during the full scan is 3.4 × 10^10^ J kg^−1^. Under the assumption that each voxel absorbs an equal amount of radiation, this equates to a dose of 1.3 × 10^6^ J kg^−1^ per voxel.

### 2.3. Simulated sample

To test the overall functioning of the analysis, we generated data from a sample of 20 × 20 × 20 voxels and placed a Gaussian ODF in each voxel. That is, we defined a mean orientation *g*_μ_ and calculated the probability for each orientation the angular distance *dg* [see Appendix *B*[App appb] equation (33)] from *g*_μ_: 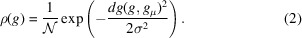
The normalization 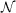 was adjusted so that 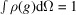 [volume element dΩ defined in equation (19)]. Standard deviations (σ) were set to 40°. These distributions were then mimicked by HSHs of order *n* = 12 to facilitate rotations in the laboratory frame. The sample was generated with stripes of equal distributions in the *x* direction and random orientations along the other axes. We generated images according to equation (26) with a BaCO_3_ structure factor and produced 108 projections for 4 tilt angles from 0 to 45° and rotation angles in an equidistant manner as described above. Data were renormalized to a maximum number of 200 counts per bin, then Poisson noise was added to simulate the conditions of a typical measurement.

### 2.4. Texture tomography inversion

The data were regrouped into 120 azimuthal and 50 *q*-bins over a range from 10 to 35 nm^−1^ using the *PyFAI* package (Ashiotis *et al.*, 2015 ▸). The *q*-regions between the Bragg peaks were masked before refinement. To correct for deviations from the true rotation center of the sample, the individual projections were aligned using the tomographic self-consistency method (Guizar-Sicairos *et al.*, 2015 ▸). The regular grid of 140 × 140 × 180 voxels was constructed, of which 26 425 voxels were identified as sample based on the scattering intensity in the SAXS region (*q* 0.5–1 nm^−1^). Gaussian beam intensities were calculated as described in Section 3.2, and voxels receiving <1% of the maximum intensity were excluded from the simulation of the respective diffraction pattern. BaCO_3_ single-crystal diffraction patterns were simulated using a published witherite crystal structure (Holl *et al.*, 2000 ▸) with the lattice parameters *a* = 5.3072 Å, *b* = 8.8928 Å, *c* = 6.4245 Å, and the space group *Pmcn*. Rotation symmetry generators for fundamental zone and HSH symmetrization were 2_001_ and 2_010_ as for the 222-point group.

All reconstructions were carried out on a standard compute node, equipped with a double CPU setup (2× AMD Epyc 7662 64 core) and 2 TB of RAM. The *TexTOM* reconstruction code is written in Python, using *numpy* and *numba* for just-in-time compilation and parallelization of the essential parts of the code. No further code optimization has been carried out and we expect that GPU portation of the code will enable a further massive speed-up of the computations, owing to the small memory footprint of the actual inversion problem. Further information on the reconstruction times are given in Table 1 ▸. A damping factor of *k* = 2 was used to ensure positivity of the ODF as further described in Section 3.7, equation (28).

## 3. Texture tomography

### 3.1. Overview

The general idea of *TexTOM* is to provide a reconstruction scheme to extract quantitative, local crystallographic texture information in 3D from a series of X-ray diffraction patterns of a sample containing polycrystalline domains in various orientations.

**Figure 1:**
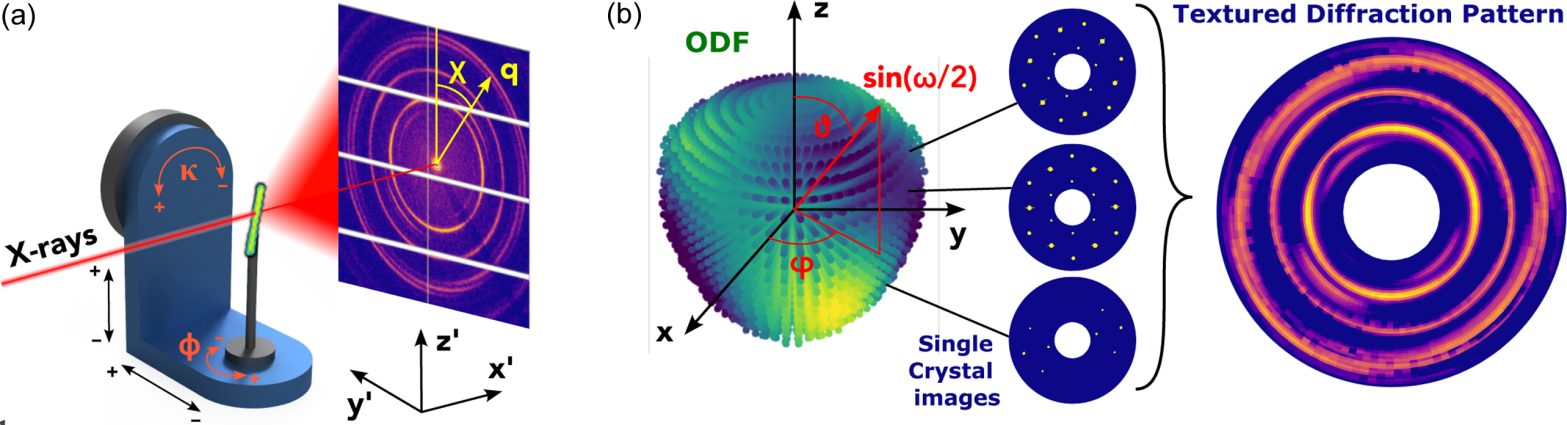
Schematics of acquiring experimental and simulated diffraction patterns. (*a*) The sample is raster-scanned using a focused X-ray beam in the *y*′/*z*′ direction for various rotation (ϕ) and tilt (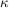) angles. At each point, a full diffraction pattern is collected, parametrized by the momentum transfer *q* and the azimuthal component χ of diffraction. (*b*) A simulated diffraction pattern originates from an ODF and a crystal structure. The ODF is parametrized by orientations of the three angles (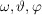) which describe axis–angle rotations in the sample CS (*x*, *y*, *z*). The ODF shown is color-coded so that brighter colors mean higher probability of the respective orientation. Each crystal orientation yields a different single-crystal diffraction pattern and the resulting image is the sum over all of them weighted by the ODF.

A brief overview of the experimental configuration is given in Fig. 1 ▸(*a*). A sample is mounted on a goniometric stage that enables scanning (*y*′/*z*′ direction), rotation and tilting (ϕ/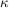) of the sample. The sample is raster scanned with a focused X-ray beam and 2D diffraction patterns are collected at each scan position. This procedure is repeated for various tilt and rotation angles, in strict analogy to tensor tomography (Liebi *et al.*, 2015 ▸).

The reconstructed quantity is a 3D ODF, representing the local arrangement of the crystallites via probabilities of all unambiguous orientations. Given that the structure of a single crystal is known, we can simulate the diffraction pattern of a polycrystalline sample by an ODF-weighted sum over the diffraction patterns of all crystal orientations. The challenge in this approach is that a faithful reconstruction of the diffraction pattern requires a high angular resolution, to ensure not to miss contributions from Bragg reflections from orientations between the sampling points. Summing over the single-crystal patterns for all orientations can therefore become computationally expensive when it comes to parameter optimization of large samples.

We therefore choose to build up our diffraction patterns from a basis set of elementary images, further labeled *diffractlets*, which originate in a set of orthogonal functions used to model the ODF. The basis is given by a series expansion of hyperspherical harmonics (HSH) (Mason & Schuh, 2008 ▸), similar to a Fourier expansion in 1D and spherical harmonics in 2D. This model therefore encodes the ODF in expansion coefficients, which are optimized in an iterative process. The calculation is roughly divided into the following steps:

(*a*) The sample is partitioned into cubic voxels, whose dimensions correspond to the step size of the raster scan.

(*b*) Each voxel contains an ODF, given by a set of coefficients of the HSH expansion.

(*c*) We calculate the projected expansion coefficients by summing them over all voxels, weighted by the respective beam intensity.

(*d*) A diffraction pattern is simulated by summing over the *diffractlets* multiplied by the projected coefficients.

(*e*) The discrepancy between simulation and data is calculated by an error metric, which is minimized iteratively by optimizing the voxel-specific coefficients.

### 3.2. Sample translations and rotations

The sample center is located at **x**′_0_ in the laboratory coordinate system (CS) as shown in Fig. 1 ▸ and we call **x**′_*i*_ the position of voxel *i* in laboratory coordinates. We assign a central voxel around which the rotations are performed, located at **x**′_0_, and surround it by a cubic lattice with edge length Δ*x*, which is the distance between two neighboring measurements. When we rotate the sample by the angles ϕ and 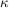, we can calculate the position of voxel *i* in the sample CS with origin at **x**′_0_ by 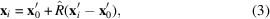
using the rotation matrix for Euler angle rotations around *z*′ and *y*′:
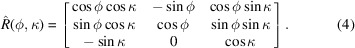
Rotating the sample mathematically comprises the challenge of rotating all voxel positions and interpolating their values on a new grid of coordinates. This process is slow and prone to produce numerical errors. For calculating the relative positions of sample and X-ray beam, we therefore keep the sample CS static and rotate the function describing the beam by the transposed rotation matrix 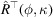. In addition, we have to include translations of the sample within the *y*′*z*′ plane (given by displacement indices *t_y_*, *t_z_* and voxel size Δ*x*). In the sample CS the translation of the beam is the negative sample displacement in the laboratory CS.

To calculate the beam path, we define two points traversed by the beam in sample coordinates: **t** is the point, where the beam traverses the *y*′*z*′ plane: 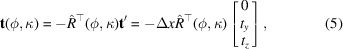
**b** is another point on the beam path, resulting from adding the beam direction unit vector 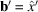: 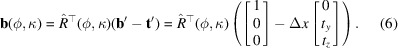
The beam intensity *B*(**x**_*i*_) at each voxel position is calculated from the cumulative distribution function of the beam profile (*e.g.* Gaussian) as a function of the normal distance *w* between the beam axis and voxel center. Note that any kind of experimentally determined beam profile can be used here. Using 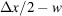 as an argument gives the intensity up to the voxel border, assuming that the beam width is equal to or smaller than the voxel. For a Gaussian beam profile with standard deviation σ, this gives 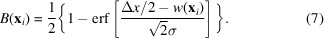
The distance *w*(**x**_*i*_) is calculated from the normal distance of the line traversing points **t** and **b** and the voxel center **x**_*i*_: 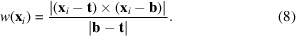
In order to account for the orientation-dependent absorption of the diffracted beam in the sample, an implementation of the absorption correction outlined by Grünewald *et al.* (2023 ▸) can be used. Due to the low absorption of the samples employed in this study, it was not implemented here.

### 3.3. Single-crystal diffraction patterns

The diffraction pattern of a single crystal *I*_sc_(**q**) is calculated from all the atom positions **R**_*j*_ from the structure factor *S*(**q**). 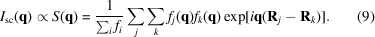
We use atomic form factors *f*(**q**) as tabulated in Henke *et al.* (1993 ▸).

The crystal is created from a known crystal structure and the unit cell is repeated in 3D to resemble the expected crystal size. We would like to note that the interface is open to accept other input sources for *S*(**q**) such as *Discus* (Proffen & Neder, 1997 ▸) in order to provide a more detailed modeling of the crystalline parameters in the future. The **q** vectors corresponding to the X-ray wavelength λ and the experimental setup are calculated using the surface of the Ewald sphere (Grünewald *et al.*, 2016 ▸). With the beam oriented along 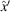 (see Fig. 1 ▸), the origin of the sphere in reciprocal space is at 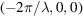 and its radius is 2π/λ. We express these points in terms of the momentum exchange 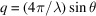 and the angle in the detector plane χ:
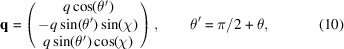
with *q* and χ corresponding to the polar coordinates on the detector, defined in Fig. 1 ▸.

In the construction of textured diffraction patterns, it is necessary to compute the contribution from each unambiguous orientation of the crystal. The diffraction pattern 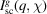 of a crystal in orientation *g* is calculated by rotating all atom positions and using the structure factor [equation (9)].

### 3.4. Orientation distribution functions

An ODF ρ(*g*) assigns a probability to a rotation *g* from a reference orientation and is expedient to describe properties of an ensemble of objects with different orientations. We use it to model the scattering intensity *I*(*q*, χ) produced by an ensemble of crystallites, assuming all of them have the same crystal structure in different orientations. The ODF connects single-crystal diffraction patterns 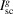 to an image resulting from an ensemble by an integration over all crystallite orientations *g*: 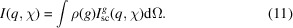


#### 3.4.1. Hyperspherical harmonic expansion

We model the ODF as a series expansion of hyperspherical harmonics (HSH) 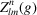 [see the Appendices[App appa][App appb][App appc] and the work by Mason (2009 ▸)]. These are complex functions that are naturally written using the axis–angle rotation parametrization. We use these rotations to describe crystal orientations with respect to the fixed sample CS. The axis–angle parameterization expresses a 3D rotation using a single rotation by an angle 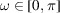 around a unit vector axis 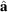, which we define by polar angle 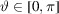 and azimuthal angle 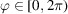: 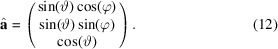
The volume element for this parametrization is given by
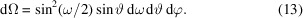
Since the HSHs form an orthonormal basis for functions of rotations, it is possible to construct an ODF as a linear combination of HSHs, where 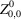 provides the probability mass and higher-order HSHs redistribute this probability mass as a function of *g*. The total ODF depends on a set of complex complex coefficients 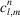 as
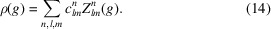
Furthermore, the ODF of a rotated sample can also be written in the form of equation (20) with a different set of coefficients. The transformation of the coefficients depends on the rotation to which the sample is subject. This is described by the matrix 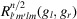 (Mason & Schuh, 2009 ▸), which allows one to write the effect of a rotation of an HSH 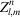 as a linear combination of other HSHs 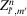 of the same order *n* (see Appendix *A*[App appa]):
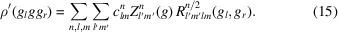
The variable *g* of the initial ODF defined in equation (20) is related to the variable 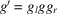 of the rotated ODF, where *g_r_* is one of the point symmetry operations of the crystal and *g_l_* is a rotation of the sample in the laboratory frame. This rotation *g_l_* will be used to simulate sample rotations in the course of the tomography experiment. The other rotation *g_r_* will always be the null rotation *g*_0_.

#### 3.4.2. Symmetrized hyperspherical harmonics

HSHs, as defined in equation (35), are complex-valued functions and require certain combinations of complex coefficients to produce a real-valued ODF. We therefore use symmetrized hyperspherical harmonics (sHSHs) 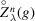, which obey this constraint by definition. The sHSHs, as functions of rotations, can also be written as linear combinations of the HSHs of the same order *n*: 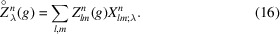
The symmetrization procedure to obtain 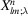 is outlined by Mason & Schuh (2008 ▸). This strategy additionally allows selection of sHSHs with the same proper point group symmetry as the crystal structure. This reduces the number of adjustable parameters, and the crystal orientations that need to be evaluated are limited to the fundamental zone of the point group (Heinz & Neumann, 1991 ▸). The matrix for the effect of a single rotation on the coefficients 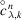 of the expansion over the sHSHs is then given by
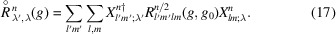


### 3.5. Full forward model

**Figure 2:**
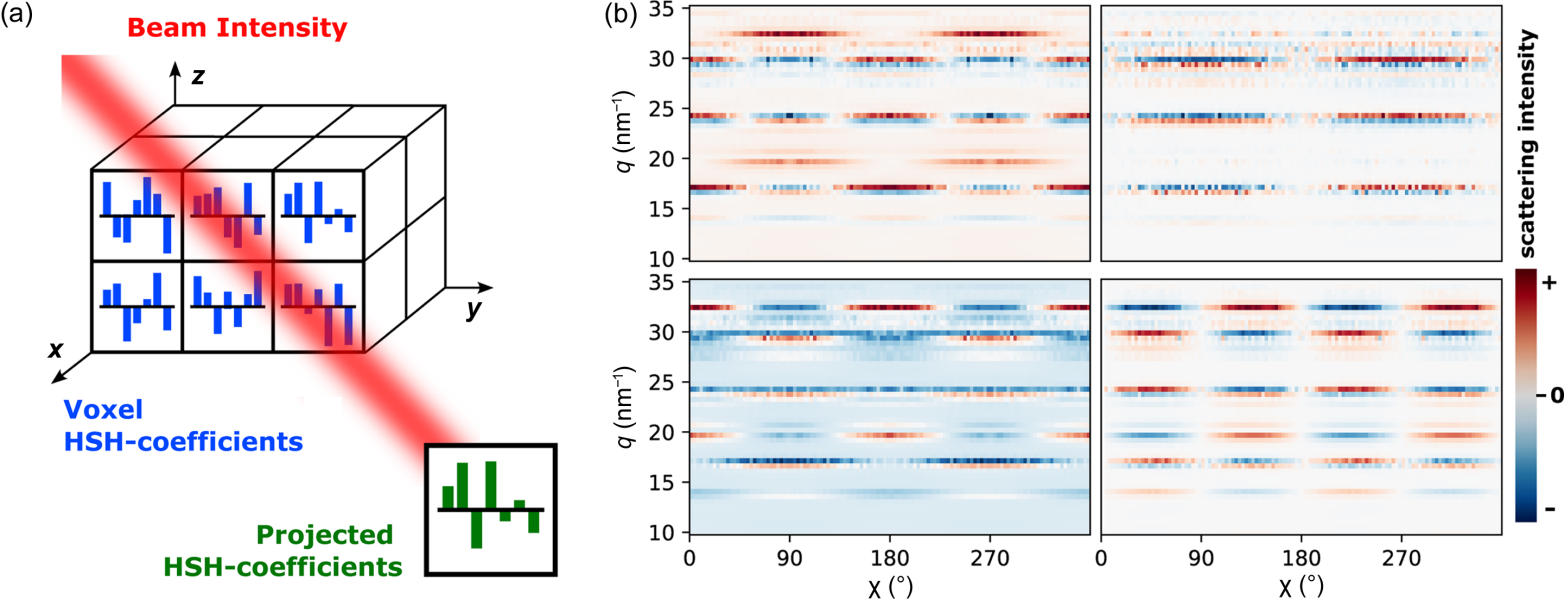
(*a*) A sample with HSH expansion coefficients in each voxel, represented by bar diagrams. These are weighted by the beam intensity for a given configuration and result in a projection of these coefficients. A summation of *diffractlets* weighted by these coefficients results in a diffraction pattern. (*b*) Selection of *diffractlets* on the order of four sHSHs with a BaCO_3_ structure factor.

Let us first derive the forward model for a sample made of a single voxel, located in **x**_1_. We can directly use the sHSH model above to calculate the diffraction patterns expected in that case: let us expand the ODF in equation (14) using sHSHs, the expected scattering intensity in any reciprocal space coordinates *q* and χ then reads 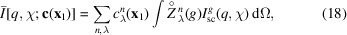
where 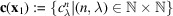 is the set of sHSH coefficients associated with a single voxel. Here, we isolate the contribution to the diffraction pattern by a single sHSH, further labeled *diffractlet* [see Fig. 2 ▸(*b*)], which reads 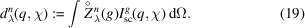
Obviously, any realistic sample should be defined over a mesh, with a series of *P* voxels located in 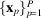. It is then natural to associate any voxel *p* with its specific series of sHSH coefficients, hence resulting in a set of parameters in the model that reads 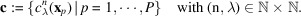
In order to account for the effect of the tomographic measurement, we introduce another index: 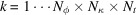, which refers to a given sample rotation ϕ, tilt 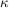 and translation **t**. The total diffraction pattern (*i.e.* the expected measurement) is a weighted sum of the contribution of each voxel in the sample, with the weights computed from the local beam intensity *B_k_*(**x**_*p*_), as given by equation (7) [illustrated in Fig. 2 ▸(*a*)]. For a single measurement *k*, we note that most of the weights are zero as only a small fraction of the voxels in the sample are actually illuminated by the beam.

The sHSH coefficients 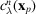 are agnostic to any rotation and tilt in the sample and we need to explicitly account for it in the forward model. To that end, we introduce first the sample rotation *g_k_* that is associated with the *k*th tomographic measurement. Then we use the rotation matrix given by equation (23) to define the corresponding *rotated diffractlets*: 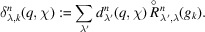
It is then straightforward to define the expected intensity for the *k*th tomographic measurement 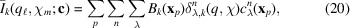
where 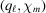 defines the measurement mesh in the reciprocal space coordinates. Clearly, this relation is a linear model that we can write in a convenient matrix–vector form: (i) first, we wrap the measurement-related indices 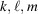 into a single index 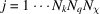; then (ii) we use a single index ν in place of the three model-related parameters *p*, *n* and λ. The relation equation (26) now reads 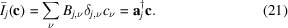
and if we stack the expected measurements 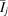 into a single vector, we obtain 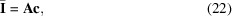
where **A** is the texture–tomography matrix that can be pre-computed before we perform the iterative inversion.

### 3.6. *TexTOM* reconstruction strategy

We use a standard quadratic metric 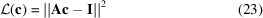
to compute the discrepancy between the output of the forward model [equation (28)] and the texture–tomographic experimental measurements 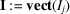. The reconstruction is defined as the minimization of the following constrained least-squares criterion 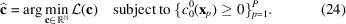
We note that the positivity constraint over the zero-order sHSH coefficients is required to produce physically meaningful results. This constraint can be fulfilled by projecting any negative 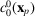 to zero after each update of the following gradient-base iteration 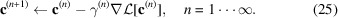
The gradient of the least-square function 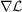 is easily derived from equation (29): 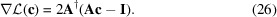
The step size γ^*n*^ is adjusted in each iteration with a *backtracking technique* to ensure a strict decrease in the criterion (Bertsekas, 1999 ▸, p. 29). We note that the fitting function [equation (29)] is ‘strictly convex’ whenever **A** is a full-column rank matrix (which is expected to be the case here). In this context, the solution of the constrained optimization problem [equation (30)] exists and is unique (Bertsekas, 1999 ▸, prop. 1.1.2), and the convergence of the iteration [equation (31)] towards 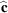 is granted, whatever the initial-guess **c**^0^.

The convergence of the iteration is monitored via the norm of the gradient, and we stop the iterative reconstruction when 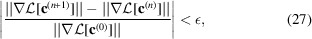
where ε is a predefined parameter (typically set to 10^−3^).

### 3.7. Data post-processing

A known limitation of the harmonic expansion approach is that it is hard to ensure positivity of the ODF (Hielscher & Schaeben, 2008 ▸), in particular without explicitly calculating the ODF in every iteration, which can be computationally extremely costly for large samples. We therefore post-process our data using a kernel that ensures non-negativity (Mason & Johnson, 2013 ▸). This means we modify the obtained coefficients by a prefactor *K* that depends on the order *n*, the highest used order *N* and an exponent *k*, whose value is empirically chosen to be between 1 and 2 according to the situation. 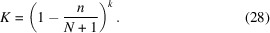
It was observed however that this kernel slightly spreads out the ODF, therefore the standard deviations presented are possibly over-estimated.

## 4. Results and discussion

### 4.1. Reconstruction of simulated data

**Figure 3:**
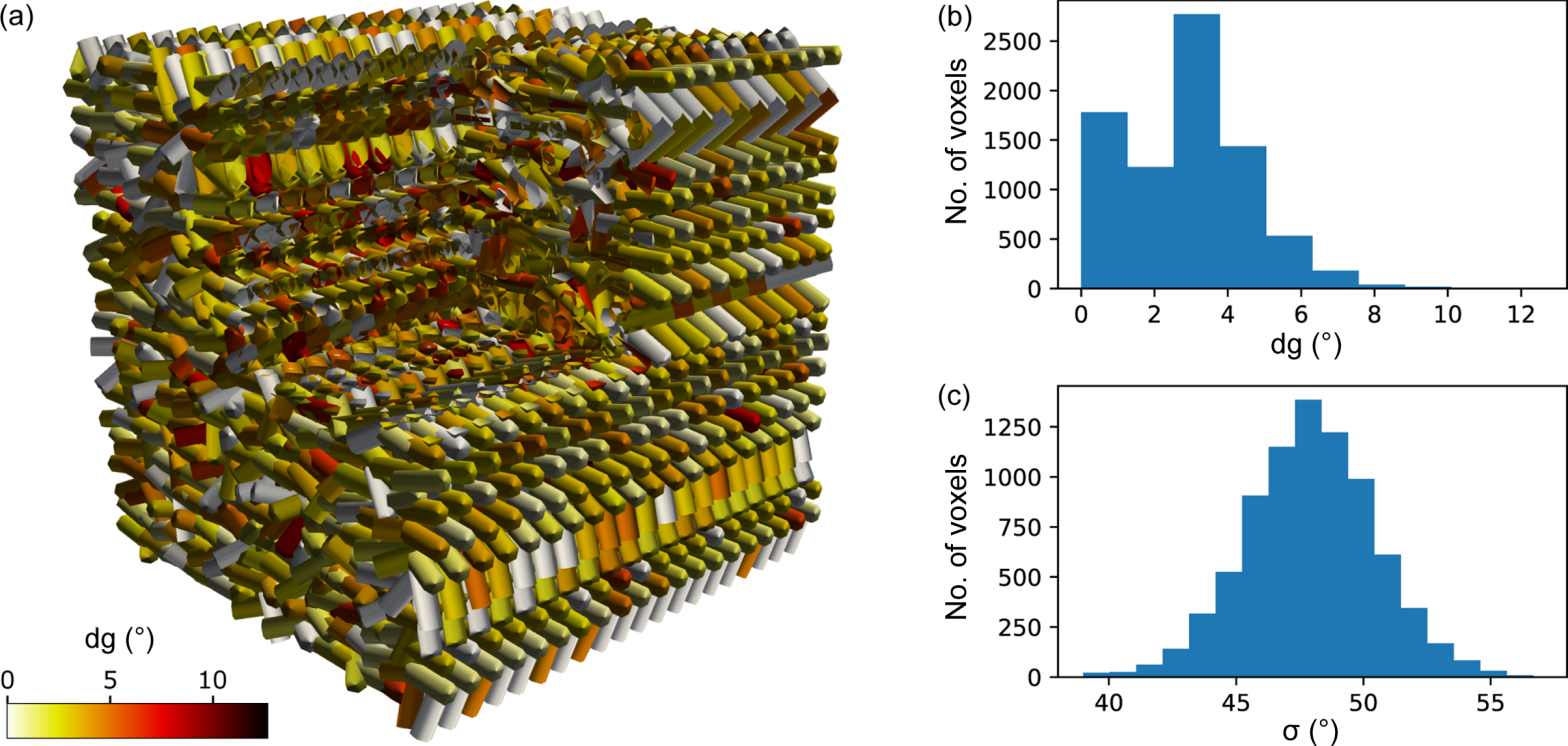
(*a*) *TexTOM* reconstruction of the simulated sample for testing the reconstruction algorithm. The sticks represent the reconstructed preferred orientation of the crystal *c* axis in each voxel, color coded by the angular deviation *dg* from the simulation. A corner was cut out to show the interior of the sample. (*b*) Histogram of *dg* for the same sample. The distribution of the standard deviations is shown in (*c*).

The reconstruction of ODFs of the simulated sample was performed in two steps: first is the retrieval of the mean orientation using an HSH-expansion cut at the lowest order, second is the estimation of the variance by including higher orders as necessary. Fig. 3 ▸ presents a summary of the inversion results: Fig. 3 ▸(*a*) shows a cut-off view of the reconstruction. Note that this representation shows only the orientation of one crystal axis and thus does not represent the full texture information. The color scale is the angular distance metric (see Appendix *B*[App appb]) between the simulation and the reconstruction, which is shown as a histogram in Fig. 3 ▸(*b*). The deviations are distributed around an average of 2.8° and show no clear spatial distribution at the interface between differently oriented layers of the sample. The irregular shape in this histogram is connected to the distribution of sampling points in orientation space, which was constructed with an angular resolution of 3°. This part of the reconstruction was done with the expansion truncated at order 4, where the point group 222 possesses 10 sHSHs, and demonstrates that the lowest order already suffices for estimating the most likely orientations.

An expansion up to order 8 was used to estimate the standard deviations (sigma) of the ODFs. As discussed in Section 3.7, our current model overestimates the spread of the distribution as a consequence of ensuring non-negativity of the ODF. We find σ distributed around 47.9° with a standard deviation of 2.6°, as shown in Fig. 3 ▸(*c*).

### 4.2. Biomorphs

**Figure 4:**
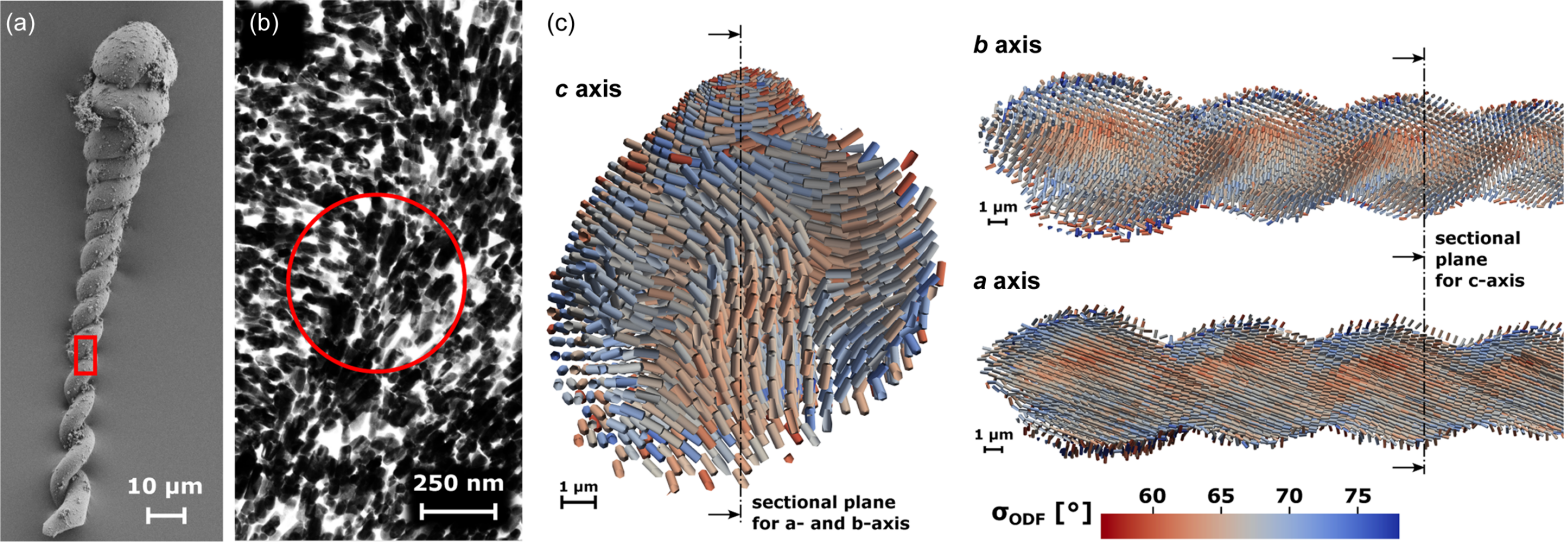
(*a*) SEM image of a helicoidal silica biomorph. (*b*) Arrangement of BaCO_3_ nanorods (black) in amorphous silica (white) as observed by TEM. For reference, the size of the region shown is given by the red rectangle in (*a*). The red circle corresponds to the dimension of the X-ray beam. (*c*) *TexTOM* reconstructions of a 60 µm-long piece of a helix. Sticks represent the preferred orientation of the indicated crystal axes. The volume is cut in transverse (*c* axis) and longitudinal (*a* and *b* axes) directions, thus showing the interior of the sample. Images were produced using *Paraview* (Ahrens *et al.*, 2005 ▸).

In order to test the performance of *TexTOM* on experimental data, a dataset of silica biomorph was collected. The sample consisted of a helicoidal silica–witherite biomorph of 60 µm length and 15 µm diameter. An exemplary SEM image can be observed in Fig. 4 ▸(*a*). The arrangement of BaCO_3_ nanorods of about 20 × 100 nm embedded in an amorphous silica matrix is shown in the TEM image [Fig. 4 ▸(*b*)]. Different morphologies can be obtained by varying the local synthesis conditions (Noorduin *et al.*, 2013 ▸; Kellermeier *et al.*, 2012 ▸). In the context of this manuscript, no in-depth analysis of different growth conditions or their texture has been carried out. Shown is the data server as a sample to demonstrate the performance of *TexTOM*.

**Figure 5:**
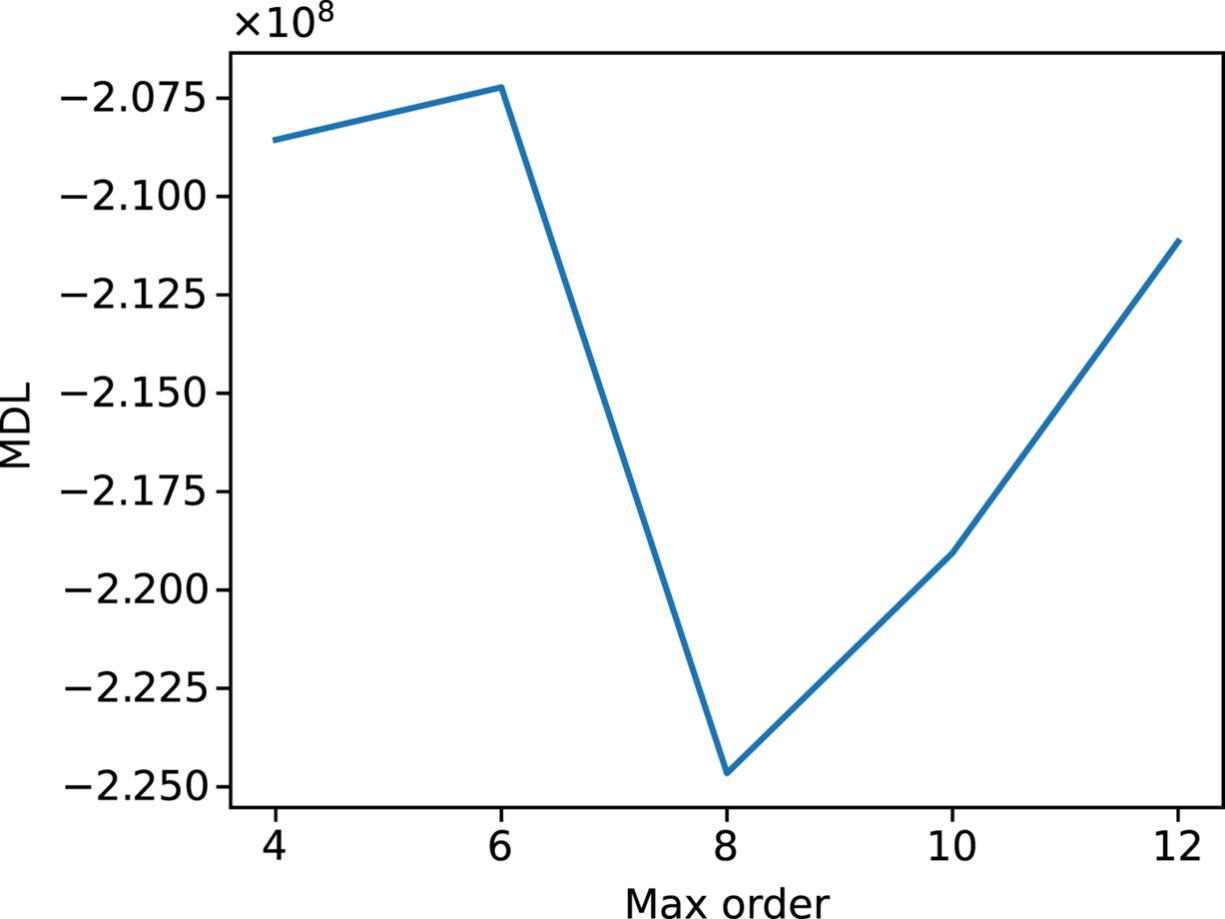
Plot of the MDL for the reconstruction of the helicoidal biomorph as a function of the HSH order *n*.

A dataset containing 260 projections was collected, with an equal angular sampling in ϕ and 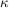 based on the reasoning of Liebi *et al.* (2018 ▸) to create a ‘gold standard’ measurement. From the reconstructions, the most likely orientation of the *a*, *b* and *c* axes was determined within in the fundamental zone and the variance was extracted as a metric for the degree of orientation/angular dispersion within each voxel and plotted as the color scale of Fig. 4 ▸(*c*). In order to facilitate the interpretation of the data, cross sections of the volume are presented in Fig. 4 ▸(*c*). The reconstructions show an angular dispersion consistent with TEM observations. The red circle indicated the beam size in comparison with the TEM image. In the slice of the *c* axis, the two distinct strands of the helix can be identified. An interesting observation is the presence of a gradient of the variance along the two helix strands, visible in the cuts of the sample showing the *a* and *b* axes of Fig. 4 ▸(*c*). These possibly correspond to a core-skin architecture in the arrangement of the nanorods. This could be caused by local concentration and pH gradients during the synthesis. A more detailed study, encompassing an in-depth, comparative analysis of different morphologies is envisaged for the future. The reconstruction strategy (described in more detail in the Materials and methods[Sec sec2]) is split into two major parts, one is the precalculation of single-crystal diffraction patterns and the beam trajectories corresponding to the scan. The rational here is that these time-consuming steps can be re-used for different reconstructions. The actual reconstructions are carried out with sequentially increasing order, which allows the selection of an optimal order via a ‘minimum description length’ (MDL) criterion (Hansen & Yu, 2001 ▸). In our case, the MDL criterion is clearly selecting the order 8 (see Fig. 5 ▸). A more detailed account of the timing is given in Table 1 ▸.

### 4.3. Benchmarking

**Figure 6:**
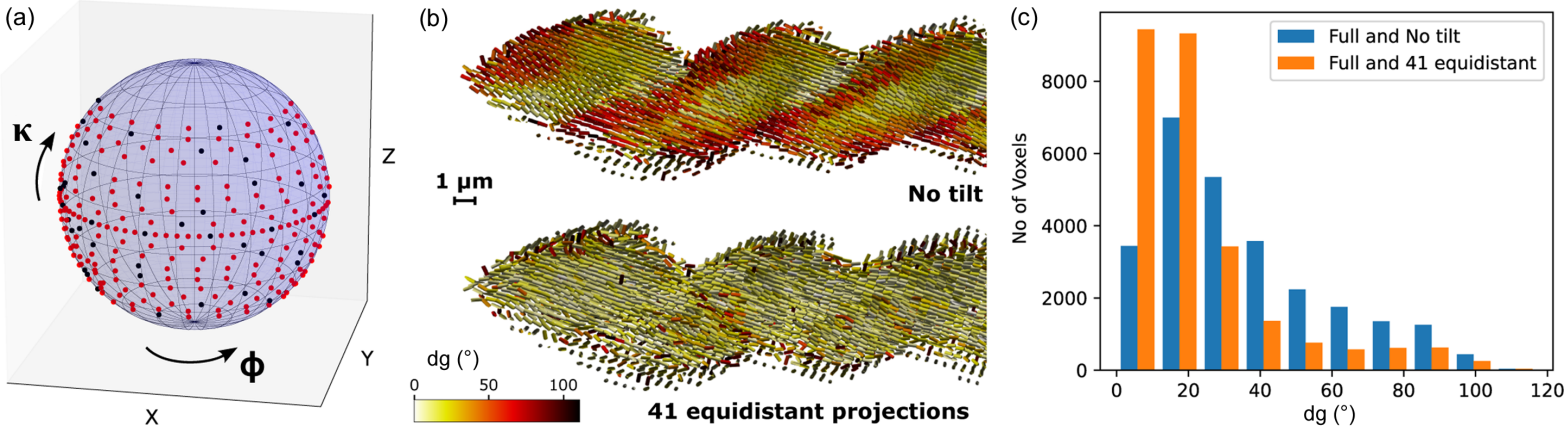
Comparison of reconstructions using all 260 projections and either 41 projections with κ = 0 or 41 projections with an equidistant distribution over orientation space. (*a*) Distribution of the 260 (red) and 41 (black) equidistant angles on the spherical CS. (*b*) Distribution of the angular deviations *dg* from the full reconstruction over the interior of the sample, using the same sectional plane as in Fig. 4 ▸. (*c*) Distribution of *dg* for both cases.

In order to test the performance of our code and see how under-sampling affected our results, two further datasets were selected from the existing data. One being all 41 projections at 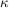 = 0 and another being 41 projections, equally sampled in ϕ and 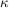 orientation space. The sampling with 41 pro­jec­tions corresponds roughly to a Nyquist–Shannon sampling (Shannon, 1949 ▸) and contains 7 equidistant ϕ angles at 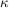 = 0 and 34 angles distributed over 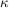 tilt angles up to 45°. Fig. 6 ▸(*a*) shows the projected distribution of all 260 angles (black) and the reduced 41 angles (red) dataset. Reconstructions were carried out and compared by calculating the angular distance between the most likely orientations for each voxel, shown in Fig. 3 ▸(*a*). The histogram in Fig. 6 ▸(*c*) shows number of voxels for each distance. It is visible that the 41 equidistant projections reproduce the orientation very well with a mean angular distance of 22° and a decrease in quality that can be expected from the sampling reduction. Here, there are no visible regions with a worse reconstruction quality, it rather adds a general noise to the results. A similar but slightly worse trend is visible for the 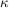 = 0 dataset. Here, the mean angular distance is 35° and the histogram [blue bars, Fig 6 ▸(*c*)] shows a higher tail at larger angles. Furthermore, the large orientation differences seem to be linked to regions where the crystallites have a wide ODF [compare red zones in top panel of Fig. 6 ▸(*a*) and blue zones in Fig. 4 ▸] hence, a weak texture, whereas voxels with a strong texture are in good agreement. This opens up the possibility for a drastic (84%) sampling reduction, which translates into faster measurement and a lower deposited dose. For the current example, the total measurement time of the 41 projections is equal to just 56 min. That is a total deposited dose of 5.3 × 10^9^ J kg^−1^ and a per-voxel dose of 2.0 × 10^5^ J kg^−1^. Together with the very fast reconstruction time (5 min) for *n* = 4 for the reduced datasets, the strategy of online reconstruction and information-driven sampling comes into reach. This strategy entails that continuous online reconstructions are carried out and that projections are added in zones of reciprocal space where the fit shows larger deviations between reconstructed and measured data.

## 5. Conclusions and outlook

In summary, this manuscript presents *TexTOM* as a new inversion framework to recover quantitative ODF information in a tomographic fashion from X-ray diffraction data using hyperspherical harmonics and derived *diffractlets*. We present a detailed description of the experiment, the forward model including the parametrization of the HSH expansion as well as our inversion strategy. We show the results of an inversion on both simulated and experimental data with submicrometre resolution and benchmark the reconstruction strategy with reduced angular sampling.

This method presents a large step forward from state-of-the-art tensor tomography methods (Liebi *et al.*, 2015 ▸; Gao *et al.*, 2019 ▸; Nielsen *et al.*, 2023 ▸) by enabling a fully quantitative reconstruction of the real-space ODF compared with the reconstruction of the position of a single SAXS or WAXS reflection. Furthermore *TexTOM* does not rely on regularization parameters and associated assumptions on local smoothness, but uses the inherent constraints given by the crystal symmetry. Due to the nature of the harmonic decomposition, the recovery of mean orientations is given by the lowest order and therefore allows very fast reconstructions, even when compared with the actual data acquisition. The estimation of the spread of the ODF requires the inclusion of higher orders and the use of a damping kernel also enables us to enforce strict positivity of each component of the ODF. This method currently overestimates the true variance and and we envisage further tuning of the model to retrieve better estimates.

The joint-optimization of several Bragg peaks also enables the concurrent refinement of multiple crystalline phases within one inversion as well as adding a strain component. For the future, the direct extraction of crystalline phase information prior to a *TexTOM* inversion via, for example, a Pawley extraction can also be envisaged, requiring less prior information on the crystalline phases present. In this way, *TexTOM* aims to reconstruct the full crystalline state tensors for each voxel with high, essentially beam-size limited resolution. One challenge arising from the wealth of information contained in the ODF is to find ways to visualize the retrieved data. Further challenges arise when trying to push the experimental resolution down into the range of 100 nm or less. Firstly, the data acquisition becomes more challenging as both the scanning and the rotation need to provide a positioning accuracy better than the target resolution. Secondly, the alignment of the data during the pre-processing is subject to equal constraints and factors such as imperfections in the tilt and rotation axis as well as the coaxiality of the scanning stages becomes more and more important and might approach the limit of current mechanical solutions. One way to overcome this challenge could be the use of non-rigid tomography approaches (Odstrcil *et al.*, 2019 ▸).

Through the efficient use of the diffraction information collected, a significant speed-up of the experiment can be expected, bringing *in situ* experiments on dynamically changing samples into reach as well as providing an avenue for measuring radiation-sensitive samples with a largely reduced deposited X-ray dose. This shall also help to keep secondary, fluoresence-induced effects (Sauer *et al.*, 2022 ▸) at bay, in particular with a potential application of this method for biomaterials. Future work will be directed towards accurately benchmarking the performance in terms of spatial, angular and multi-phase resolution with specifically designed benchmark samples. A further development is the implementation of live reconstructions and information-driven sampling to realize the quickest-possible experiments whilst achieving the desired angular and spatial resolution.

## Figures and Tables

**Table 1:** Duration *t* of different steps of *TexTOM* reconstructions and the total number of adjustable parameters *N*_par_

Pre-calculations	*t* (min)	
Single-crystal diffraction patterns	770	
Beam trajectories	5	

260 projections	*t* (min)	*N*_par_
Reconstruction *n* = 4	35	290675
Reconstruction up to *n* = 8	100	1189125
Reconstruction up to *n* = 12	305	3144575

41 projections	*t* (min)	*N*_par_
Reconstruction *n* = 4	5	290675
Reconstruction up to *n* = 8	24	1189125
Reconstruction up to *n* = 12	60	3144575

## Data Availability

The *TexTOM* code used in this publication is available via zenodo https://zenodo.org/records/12543638.

## Appendix A Equations related to hyperspherical harmonics

Definition of hyperspherical harmonics (Mason, 2009 ▸):

where  are the Gegenbauer polynomials and the  are the associated Legendre polynomials with the Condon–Shortley phase. Their integer indices are restricted to , , .

The hyperspherical harmonic rotation matrix is given by

where  is the irreproducible representative of *SO*(3)

and  being the Clebsch–Gordon coefficient for *SO*(3).

## Appendix B Angular distance between orientations

We define the distance between two orientations  via the quaternion formalism. Unit quaternions are related to the rotation angle ω and axis  by

The angular distance between two unit quaternions is then given by (Huynh, 2009 ▸)

Here,  are the quaternions corresponding to rotations , respectively.  denotes the inner product of the quaternions:

This however, does not yet take into account the crystal symmetry. To find the effective minimal distance between two orientations, we therefore rotate **q** by the symmetry generators of the point group and choose the smallest distance of all possible combinations.

## Appendix C Preferred orientation and standard deviation of ODFs

We examined the resulting ODFs from the sHSH expansion and found that the ODF truncated at the lowest order (*n* = 4 for point group ‘222’) generally shows a single maximum within the fundamental zone. We define this orientation as the mean orientation *g*_μ_ of the distribution. To calculate a standard deviation of an ODF given by a set of coefficients **c**, we rotate the distribution by −*g*_μ_ using equation (21), thus obtaining a distribution centered at the center of the fundamental zone (where ω = 0). Then we calculate the standard deviation σ in the conventional way from the rotation angle ω:

## References

[bb1] Ahrens, J., Geveci, B. & Law, C. (2005). *ParaView: An End-User Tool for Large Data Visualization*, *Visualization Handbook.* Munich: Elsevier.

[bb2] Ashiotis, G., Deschildre, A., Nawaz, Z., Wright, J. P., Karkoulis, D., Picca, F. E. & Kieffer, J. (2015). *J. Appl. Cryst.* **48**, 510–519. 10.1107/S1600576715004306 PMC4379438 25844080

[bb3] Bachmann, F., Hielscher, R. & Schaeben, H. (2010). *Solid State Phenom.* **160**, 63–68.

[bb901] Bertsekas, D. P. (1999) *Nonlinear Programming*. Athena Scientific.

[bb4] Bleuet, P., Welcomme, E., Dooryhée, E., Susini, J., Hodeau, J.-L. & Walter, P. (2008). *Nat. Mater.* **7**, 468–472. 10.1038/nmat2168 18425135

[bb5] Bunge, H.-J. (1982). *Texture Analysis in Materials Sciences.* London: Butterworth-Heinemann.

[bb6] Bunge, H.-J. (2013). *Texture Analysis in Materials Science: Mathematical Methods.* Göttingen: Elsevier.

[bb7] Bunge, H.-J. & Roberts, W. T. (1969). *J. Appl. Cryst.* **2**, 116–128.

[bb8] Cloetens, P., Ludwig, W., Baruchel, J., Van Dyck, D., Van Landuyt, J., Guigay, J. P. & Schlenker, M. (1999). *Appl. Phys. Lett.* **75**, 2912–2914.

[bb9] Dierolf, M., Menzel, A., Thibault, P., Schneider, P., Kewish, C. M., Wepf, R., Bunk, O. & Pfeiffer, F. (2010). *Nature*, **467**, 436–439. 10.1038/nature09419 20864997

[bb10] Gao, Z., Guizar-Sicairos, M., Lutz-Bueno, V., Schröter, A., Liebi, M., Rudin, M. & Georgiadis, M. (2019). *Acta Cryst.* A**75**, 223–238. 10.1107/S2053273318017394 PMC6396401 30821257

[bb11] Garcia-Ruiz, J. M. (1985). *J. Cryst. Growth*, **73**, 251–262.

[bb12] Georgiadis, M., Guizar-Sicairos, M., Zwahlen, A., Trüssel, A. J., Bunk, O., Müller, R. & Schneider, P. (2015). *Bone*, **71**, 42–52. 10.1016/j.bone.2014.10.002 25306893

[bb13] Georgiadis, M., Schroeter, A., Gao, Z., Guizar-Sicairos, M., Liebi, M., Leuze, C., McNab, J. A., Balolia, A., Veraart, J., Ades-Aron, B., Kim, S., Shepherd, T., Lee, C. H., Walczak, P., Chodankar, S., DiGiacomo, P., David, G., Augath, M., Zerbi, V., Sommer, S., Rajkovic, I., Weiss, T., Bunk, O., Yang, L., Zhang, J., Novikov, D. S., Zeineh, M., Fieremans, E. & Rudin, M. (2021). *Nat. Commun.* **12**, 2941. 10.1038/s41467-021-22719-7 PMC8134484 34011929

[bb14] Godard, P., Carbone, G., Allain, M., Mastropietro, F., Chen, G., Capello, L., Diaz, A., Metzger, T., Stangl, J. & Chamard, V. (2011). *Nat. Commun.* **2**, 568. 10.1038/ncomms1569 22127064

[bb15] Grünewald, T. A., Johannes, A., Wittig, N. K., Palle, J., Rack, A., Burghammer, M. & Birkedal, H. (2023). *IUCrJ*, **10**, 189–198. 10.1107/S2052252523000866 PMC9980387 36786504

[bb16] Grünewald, T. A., Liebi, M., Wittig, N. K., Johannes, A., Sikjaer, T., Rejnmark, L., Gao, Z., Rosenthal, M., Guizar-Sicairos, M., Birkedal, H. & Burghammer, M. (2020). *Sci. Adv.* **6**, eaba4171. 10.1126/sciadv.aba4171 PMC7292642 32582855

[bb17] Grünewald, T. A., Rennhofer, H., Tack, P., Garrevoet, J., Wermeille, D., Thompson, P., Bras, W., Vincze, L. & Lichtenegger, H. C. (2016). *Angew. Chem. Int. Ed.* **55**, 12190–12194. 10.1002/anie.201603784 27483396

[bb18] Guizar-Sicairos, M., Boon, J. J., Mader, K., Diaz, A., Menzel, A. & Bunk, O. (2015). *Optica*, ** 2**, 259.

[bb19] Hansen, M. & Yu, B. (2001). *J. Am. Stat. Assoc.* **96**, 746–774.

[bb20] Heinz, A. & Neumann, P. (1991). *Acta Cryst.* A**47**, 780–789.

[bb21] Helmbrecht, L., Tan, M., Röhrich, R., Bistervels, M. H., Kessels, B. O., Koenderink, A. F., Kahr, B. & Noorduin, W. L. (2020). *Adv. Funct. Mater.* **30**, 1908218.

[bb22] Henke, B. L., Gullikson, E. M. & Davis, J. C. (1993). *At. Data Nucl. Data Tables*, **54**, 181–342.

[bb23] Hielscher, R. & Schaeben, H. (2008). *J. Appl. Cryst.* **41**, 1024–1037.

[bb24] Holl, C. M., Smyth, J. R., Laustsen, H. M. S., Jacobsen, S. D. & Downs, R. T. (2000). *Phys. Chem. Miner.* **27**, 467–473.

[bb25] Holtus, T., Helmbrecht, L., Hendrikse, H. C., Baglai, I., Meuret, S., Adhyaksa, G. W. P., Garnett, E. C. & Noorduin, W. L. (2018). *Nat. Chem.* **10**, 740–745. 10.1038/s41557-018-0064-1 29867120

[bb26] Howells, M., Beetz, T., Chapman, H., Cui, C., Holton, J., Jacobsen, C., Kirz, J., Lima, E., Marchesini, S., Miao, H., Sayre, D., Shapiro, D., Spence, J. & Starodub, D. (2009). *J. Electron Spectrosc. Relat. Phenom.* **170**, 4–12. 10.1016/j.elspec.2008.10.008 PMC2867487 20463854

[bb27] Huynh, D. Q. (2009). *J. Math. Imaging Vis.* **35**, 155–164.

[bb28] Johannes, A., Rensberg, J., Grünewald, T. A., Schöppe, P., Ritzer, M., Rosenthal, M., Ronning, C. & Burghammer, M. (2020). *J. Appl. Cryst.* **53**, 99–106.

[bb29] Kellermeier, M., Melero–García, E., Glaab, F., Eiblmeier, J., Kienle, L., Rachel, R., Kunz, W. & García–Ruiz, J. M. (2012). *Chem. A Eur. J.* **18**, 2272–2282. 10.1002/chem.201102407 22259042

[bb30] Kocks, U. F., Tomé, C. N., Wenk, H.-R., Beaudoin, A. J. & Mecking, H. (2000). *Texture and Anisotropy: Preferred Orientations in Polycrystals and their Effect on Materials Properties*, 1st ed. Cambridge University Press.

[bb31] Liebi, M., Georgiadis, M., Kohlbrecher, J., Holler, M., Raabe, J., Usov, I., Menzel, A., Schneider, P., Bunk, O. & Guizar-Sicairos, M. (2018). *Acta Cryst.* A**74**, 12–24. 10.1107/S205327331701614X PMC5740453 29269594

[bb32] Liebi, M., Georgiadis, M., Menzel, A., Schneider, P., Kohlbrecher, J., Bunk, O. & Guizar-Sicairos, M. (2015). *Nature*, **527**, 349–352. 10.1038/nature16056 26581291

[bb33] Lutterotti, L., Matthies, S., Wenk, H.-R., Schultz, A. & Richardson, J. Jr (1997). *J. Appl. Phys.* **81**, 594–600.

[bb34] Mason, J. & Schuh, C. (2008). *Acta Mater.* **56**, 6141–6155.

[bb35] Mason, J. & Schuh, C. (2009). *Metall. Mater. Trans. A*, **40**, 2590–2602.

[bb36] Mason, J. K. (2009). PhD thesis. Massachusetts Institute of Technology, MA, USA.

[bb37] Mason, J. K. & Johnson, O. K. (2013). *J. Appl. Cryst.* **46**, 1722–1728.

[bb38] Matthies, S. & Vinel, G. W. (1982). *Phys. Status Solidi B*, **112**.

[bb39] Morawiec, A. (1990). *J. Appl. Cryst.* **23**, 374–377.

[bb40] Mürer, F. K., Chattopadhyay, B., Madathiparambil, A. S., Tekseth, K. R., Di Michiel, M., Liebi, M., Lilledahl, M. B., Olstad, K. & Breiby, D. W. (2021). *Sci. Rep.* **11**, 2144. 10.1038/s41598-020-80615-4 PMC7835348 33495539

[bb41] Nielsen, L. C., Erhart, P., Guizar-Sicairos, M. & Liebi, M. (2023). *Acta Cryst.* A**79**, 515–526. 10.1107/S205327332300863X PMC10626654 37855136

[bb42] Niese, S., Krüger, P., Kubec, A., Braun, S., Patommel, J., Schroer, C. G., Leson, A. & Zschech, E. (2014). *Opt. Express*, **22**, 20008. 10.1364/OE.22.020008 25321210

[bb43] Noorduin, W. L., Grinthal, A., Mahadevan, L. & Aizenberg, J. (2013). *Science*, **340**, 832–837. 10.1126/science.1234621 23687041

[bb44] Odstrcil, M., Holler, M., Raabe, J., Sepe, A., Sheng, X., Vignolini, S., Schroer, C. G. & Guizar-Sicairos, M. (2019). *Nat. Commun.* **10**, 2600. 10.1038/s41467-019-10670-7 PMC6565693 31197135

[bb45] Opel, J., Hecht, M., Rurack, K., Eiblmeier, J., Kunz, W., Cölfen, H. & Kellermeier, M. (2015). *Nanoscale*, **7**, 17434–17440. 10.1039/c5nr05399d 26439927

[bb46] Opel, J., Rosenbaum, L.-C., Brunner, J., Staiger, A., Zimmermanns, R., Kellermeier, M., Gaich, T., Cölfen, H. & García-Ruiz, J.-M. (2020). *J. Mater. Chem. B*, **8**, 4831–4835. 10.1039/c9tb02955a 32432609

[bb47] Opel, J., Wimmer, F. P., Kellermeier, M. & Cölfen, H. (2016). *Nanoscale Horizons*, ** 1**, 144–149. 10.1039/c5nh00094g 32260636

[bb48] Paganin, D. M. & Pelliccia, D. (2021). *Adv. Imaging Electron Phys.* **218**, 63–158.

[bb49] Poulsen, H. F., Nielsen, S. F., Lauridsen, E. M., Schmidt, S., Suter, R. M., Lienert, U., Margulies, L., Lorentzen, T. & Juul Jensen, D. (2001). *J. Appl. Cryst.* **34**, 751–756.

[bb50] Proffen, Th. & Neder, R. B. (1997). *J. Appl. Cryst.* **30**, 171–175.

[bb51] Raimondi, P., Benabderrahmane, C., Berkvens, P., Biasci, J. C., Borowiec, P., Bouteille, J.-F., Brochard, T., Brookes, N. B., Carmignani, N., Carver, L. R., Chaize, J.-M., Chavanne, J., Checchia, S., Chushkin, Y., Cianciosi, F., Di Michiel, M., Dimper, R., D’Elia, A., Einfeld, D., Ewald, F., Farvacque, L., Goirand, L., Hardy, L., Jacob, J., Jolly, L., Krisch, M., Le Bec, G., Leconte, I., Liuzzo, S. M., Maccarrone, C., Marchial, T., Martin, D., Mezouar, M., Nevo, C., Perron, T., Plouviez, E., Reichert, H., Renaud, P., Revol, J.-L., Roche, B., Scheidt, K.-B., Serriere, V., Sette, F., Susini, J., Torino, L., Versteegen, R., White, S. & Zontone, F. (2023). *Commun. Phys.* **6**, 82.

[bb52] Robinson, A. C. (1958). *On the Use of Quaternions in Simulation of Rigid-Body Motion.* Wright Air Development Center, USA.

[bb53] Roe, R. (1965). *J. Appl. Phys.* **36**, 2024–2031.

[bb54] Sauer, K., Zizak, I., Forien, J.-B., Rack, A., Scoppola, E. & Zaslansky, P. (2022). *Nat. Commun.* **13**, 7829. 10.1038/s41467-022-34247-z PMC9768145 36539409

[bb55] Schaff, F., Bech, M., Zaslansky, P., Jud, C., Liebi, M., Guizar-Sicairos, M. & Pfeiffer, F. (2015). *Nature*, **527**, 353–356. 10.1038/nature16060 26581292

[bb56] Schroer, C. G., Kuhlmann, M., Roth, S. V., Gehrke, R., Stribeck, N., Almendarez-Camarillo, A. & Lengeler, B. (2006). *Appl. Phys. Lett.* **88**, 164102.

[bb57] Shannon, C. (1949). *Proc. Inst. Radio Eng. (IRE)*, ** 37**, 10–21.

[bb58] Silva Barreto, I., Pierantoni, M., Nielsen, L. C., Hammerman, M., Diaz, A., Novak, V., Eliasson, P., Liebi, M. & Isaksson, H. (2024). *Acta Biomaterialia*, **174**, 245–257. 10.1016/j.actbio.2023.12.015 38096959

[bb59] Simons, H., King, A., Ludwig, W., Detlefs, C., Pantleon, W., Schmidt, S., Stöhr, F., Snigireva, I., Snigirev, A. & Poulsen, H. F. (2015). *Nat. Commun.* **6**, 6098. 10.1038/ncomms7098 PMC4354092 25586429

[bb60] Stock, S., De Carlo, F. & Almer, J. (2008). *J. Struct. Biol.* **161**, 144–150. 10.1016/j.jsb.2007.10.001 18006333

[bb61] Tavares, P. F., Al-Dmour, E., Andersson, Å., Cullinan, F., Jensen, B. N., Olsson, D., Olsson, D. K., Sjöström, M., Tarawneh, H., Thorin, S. & Vorozhtsov, A. (2018). *J. Synchrotron Rad.* **25**, 1291–1316. 10.1107/S1600577518008111 PMC6140400 30179168

[bb62] Wigner, E. P. (2012). *Group Theory and its Application to the Quantum Mechanics of Atomic Spectra*. New York: Academic Press.

[bb63] Williams, G. J., Pfeifer, M. A., Vartanyants, I. A. & Robinson, I. K. (2003). *Phys. Rev. Lett.* **90**, 175501. 10.1103/PhysRevLett.90.175501 12786079

